# Maternal and Neonatal Characteristics for Late Foetal Death in Latvia between 2001 and 2014: Population-Based Study

**DOI:** 10.1155/2018/2630797

**Published:** 2018-07-18

**Authors:** Irisa Zile, Inguna Ebela, Valdis Folkmanis, Ingrida Rumba Rozenfelde

**Affiliations:** ^1^Faculty of Medicine, Department of Paediatrics, University of Latvia, Raina bulv. 19, Riga LV-1586, Latvia; ^2^Department of Research, Statistics and Health Promotion, Centre for Disease Prevention and Control of Latvia, Duntes 22, k-5, Riga LV-1005, Latvia

## Abstract

**Introduction:**

Stillbirth is one of the most common adverse pregnancy outcomes worldwide. Late foetal death (LFD) rates are mostly used for international comparisons because of the large variations in stillbirth rates between countries.

**Objective:**

To examine trends in LFD (including antepartum and intrapartum) by multiple births, birth weight, and maternal age in two time periods.

**Methods:**

A retrospective cohort study was used to analyse data from the Medical Birth Register (2001–2014), divided into 2 periods of 7 years each. In total, data on 1,340 singletons were analysed. This study calculated LFD rates and rate ratios (RR).

**Results:**

The overall LFD rate showed a slight statistically significant reduction (p < 0.001) of 18% between 2001–2007 and 2008–2014. There was a slight increase in the mortality rate from multiple pregnancies (RR 1.1/1000; 95% CI 0.6-1.9). There were no major differences in the LFD rate by maternal age during the time periods.

**Conclusions:**

LFD decreased (RR 0.8/1000 births), as well as intrapartum LFD (RR 0.6/1000 births). Older maternal age influenced pregnancy outcomes, and higher LFD rates were observed in the age group ≥35 years. Substantial intrapartum stillbirths rates indicate problems with quality of intrapartum care and emergency obstetric care. Further research is needed to evaluate the strategies necessary to substantially reduce the number of stillbirths in the country.

## 1. Introduction

Stillbirth is one of the most common adverse pregnancy outcomes worldwide; over 3 million deliveries annually are stillborn [1–4]. The European Perinatal Health Monitoring data (PERISTAT) analysis shows that the average reduction of stillbirths in 2010 compared to 2004 was approximately 19% (*variations among countries to 38*%) [5, 6]. Late foetal death rates are used for international comparisons because of large stillbirth rate variations between countries [7–9].

The number of stillbirths, which were explicitly targeted in the Millennium Development Goals, has decreased more slowly than has infant mortality or mortality in children younger than 5 years. The Every Newborn Action Plan has a target of 12 or fewer stillbirths per 1000 births in every country by 2030. A total of 94 mainly high-income countries and upper-middle-income countries have already met this target, although with noticeable disparities [3].

Variations in stillbirth rates across high-income countries and large equity gaps within high-income countries persist [10]. Disadvantaged women, those with less antenatal care and those who delivered without a skilled birth attendant were at increased risk of delivering a stillbirth [3, 11, 12]. Each death is a tragic loss and causes much grief to the parents and extended family. These deaths matter to the mother and the family, to society, and to the health care system. Stillbirths are associated with public health challenges such as social inequalities, maternal obesity, and smoking [13].

Total stillbirth rates in Latvia have seen little change in the past 16 years. A slight decrease has been observed from 7.0/1000 births in 2001 to 5.7/1000 births in 2016 [14]. The aim of this study was to examine trends in late foetal death (including antepartum and intrapartum) by multiple births, birth weight, and maternal age in two time periods.

## 2. Materials and Methods

The study was a population-based registry study. Data were obtained from the Medical Birth Registry. All births in Latvia (including stillbirths) are compulsorily reported to the registry, and notification is made by standardized medical record forms used by all maternity units across the country. Late foetal deaths were defined as stillbirths occurring after 28 completed weeks of gestation and weighing at least 500 g. In total, the data on 1,340 LFD cases were analysed from 2001 to 2014, divided into 2 periods of 7 years each (2001-2007 and 2008-2014).

Descriptive statistics for all of the continuous variables (maternal age, foetal birth weight, and gestational week) are reported as medians, indicating the 25th and 75th percentiles. Categorical data are reported as percentages and 95% confidence intervals (CI). The categorical variables were compared using chi-square tests. P values < 0.05 were considered statistically significant.

The study described and compared maternal and antenatal care factors (including complete care, lack of antenatal care, and delayed antenatal care, i.e., the first visit after the 12th gestational week (GW)) and certain complications over the 2 time periods. Antenatal care quality was defined by three groups: without care (cases with LFD when mother has not been registered to gynaecology for antenatal care and therefore did not receive any antenatal checks), complete care (first antenatal visit till 12th week of pregnancy and totally 7 obligatory antenatal visits with all necessary antenatal checks and tests as per guidelines (blood, urine tests; ultrasound screening; genetic tests; etc.), and incomplete (one or more conditions are missing, e.g., late first antenatal visit or less antenatal visits or checks).

Maternal age was coded as follows: ≤19 years, 20-34 years, and ≥35 years.

Late foetal death rates were calculated per 1000 total births in each time period. Time trends were analysed by calculating rate ratios (RR) with 95% confidence intervals (CI), and rates in 2001-2007 were compared with those in 2008-2014.

The study was conducted with the approval of the Ethics Committee of the University of Latvia.

## 3. Results

A total of 74% of all stillbirths from 2001 to 2014 were late foetal deaths (n = 1,340). The median maternal age in the surveyed population was 28 years (*23-33*), the median birth weight was 2380 g (*1620-3100*), and the median gestational week was 36 (*32-39*). There were no differences in medians by time periods. 55% (n = 732) was preterm birth. More than half were antepartum stillbirths, there was an increase (p < 0.001) between the 2 time periods. Intrapartum stillbirths make quite a large proportion although decrease by 6.1 percentage points (p < 0.001) was observed over time (22.8% to 16.7%). There were no changes detected in LFD by gestational age and birth weight (Table 1).

An analysis of antenatal care factors that may be associated with adverse pregnancy outcomes found that a total of 29.0% (95% CI 26.7-31.5) of stillbirths occurred in cases of late registration for antenatal care (after 12 GW). However, in those time periods, a decrease of 13 percentage points was observed (p < 0.001). In comparing antenatal care quality at 2 time points, there was also an observed decrease in the proportion of mothers who were without antenatal care—from 18.3% (95% CI 15.7-21.2) to 10.6% (95% CI 8.3-13.2) (p < 0.001). There were no statistically significant changes by incomplete antenatal care (17.1% (95% CI 14.5-20.1) to 15.6% (95% CI 12.8-18.6)), but complete antenatal care grew from 64.5% to 73.8% (p < 0.001). Intrauterine growth restriction detected antenatally was observed in 3.8% (n = 51), pregnancy-induced hypertension 5.5 (n = 74), preeclampsia 6.5% (n = 87), and placenta abruption 5.7% (n = 77) (Table 1).

Smoking during pregnancy was observed in a total of 20.3% (95% CI 18.2-22.5) of cases, and a slight decrease by 3.8 percentage points in smoking was observed over time; however, this difference was not statistically significant (Table 1).

Total numbers of births, live births, and LFD rates in different study time points are given in Table 2. Totally an average is 20,000 births a year. The overall late foetal death rate showed a slightly statistically significant reduction (*p* <* 0.001*) of 18% between 2001-2007 and 2008-2014. Decrease was observed also in intrapartum LFD (RR 0.6/1000 births). There were no major differences in the late foetal death rate by maternal age during the time periods. A more substantial reduction was observed in the age group ≥35 years (p < 0.001).

## 4. Discussion

Nearly 2.6 million stillbirths occur globally each year, most of which are thought to be preventable. The majority of these deaths occurred in developing countries. About half of all stillbirths occur in the intrapartum period, representing the greatest time of risk [1].

The results of the present study showed that an average of 80% of stillbirths are antepartum deaths. Our study findings indicate an indirect association between late and inadequate antenatal care and stillbirths. Our previous studies observed that the single largest risk factor for antepartum stillbirth is foetal growth restriction [15]. Preventive strategies need to focus on improving antenatal detection of foetal growth restriction [16, 17].

The intrapartum death rate of a country is reflective of the care received by mothers and babies in labour and rate higher than 10% indicates problems with obstetric care quality [17]. The number of intrapartum foetal deaths that occur in high-income countries on average is 0.3–0.7/1000 births [3, 4]. Our study showed higher intrapartum LFD rate although slightly decreased till 0.7/1000 in the second time point. Intrapartum stillbirths are largely preventable with quality intrapartum care, including prompt recognition and management of intrapartum complications [17]. Therefore antenatal care also plays a vital role in the management of a woman's health during pregnancy, and women who have not been registered to antenatal care are at an increased risk of intrapartum stillbirth too.

This study found that a very high proportion of LFD was related to a late first antenatal visit or incomplete care. Nevertheless, a positive decreasing tendency for late antenatal visits has been observed in the study period, from 2001 to 2014. High proportion was found for LFD cases and lack of antenatal care, an average 15%. According to statistical data in the general population in 2016, 0.7% of live births occurred without antenatal care, but the rate of stillbirths that occurred without prenatal care was 8 times higher, at 6.2% [18]. We have limited information in the Medical Birth Register about maternal smoking habits, but the study data indicate that the proportion of maternal smoking related to LFD was an average of 20%. Smoking during pregnancy showed a slight but nonsignificant decrease, from 22% to 18%. Statistical data in Latvia shows that maternal smoking related to live births was 7.6% and 9.3% for stillbirths in 2016 [18]. Indisputably, prenatal care plays an important role in the monitoring and control of both sociodemographic and lifestyle factors, which may contribute to poor pregnancy outcomes including stillbirths [3, 11, 13, 19].

As shown in the literature, the number of neonatal and infant deaths declined more rapidly than the number of stillbirths [1–3, 20]. Foetal and neonatal mortality rates are highly sensitive to inclusion criteria for threshold gestational age and birth weight, especially in comparison to other countries [7, 9]. However, it is no less important to obtain national data and trend analysis within the country because perinatal, foetal, and neonatal mortality statistics are also important to show the development of the health care system. Our country has limited epidemiological studies about stillbirths and late foetal death. For this reason, our research aim was to evaluate trends in late foetal death (including antepartum and intrapartum period) by multiple births, birth weight, and maternal age in two time periods to better understand and obtain more population-based data on this issue.

During the study period of 2001-2014, the overall late foetal death rate declined by 18%. Similar findings were obtained in the PERISTAT data analysis; between 2004 and 2010, stillbirths declined by 17%, with a range from 1% to 39% by country [6]. The perinatal health monitoring system shows that the foetal mortality rates at or after 28 weeks of gestation ranged from lows under 2.0 per 1000 live births and stillbirths in the Czech Republic and Iceland to 4.0 or more per 1000 live births in countries such as France and Latvia [5].

A survey using the PERISTAT data indicated that stillbirth rates in European countries declined in all gestational age subgroups. Declines were lower for stillbirths at 28-31 weeks (12%) than at 32-36 weeks (19%) and 37weeks and over (18%) [6]. There were no changes by LFD within gestational age groups by time period in our study. The high proportion of LFD was from term births (45%). These results underscore the importance of a focus on improving outcomes across the gestational age spectrum.

The study results show that LFD rates were higher in multiple births and in the maternal age group ≥35 years, although a more rapid decrease of 15% was observed in that age group in the 2 time periods. Other study results that analysed more risk factors indicated that LFD rates were increased in women who were 35 years or older [3, 8, 12, 21].

In recent years, the health of mothers and children in Latvia has been receiving increasing attention; thus, different solutions for improving the situation have been closely evaluated. Maternal and child health improvement and the reduction of mortality rates are also two of the objectives stated in the “Public Health Strategy for 2011-2017" [22] and the project document “Maternal and Child Health Improvement Plan 2018-2020" [23], developed by the Ministry of Health. The Action Plan also foresees changes in the legislative documents in screening policies, and improvements are being made in the implementation of perinatal audits in clinical practice and at the national level [23]. Quality of care includes the judgement to determine which women are at risk and require interventions. However, in addition to the quality of obstetric care, the timeliness of providing obstetric care is critical, especially to save the foetus. Tools such as perinatal audits have been shown to improve the quality of facility care and to reduce stillbirth [24, 25]. Substantial proportion of intrapartum stillbirths (higher than 10%) are preventable with quality intrapartum care and emergency obstetric care can make the greatest impact on stillbirth rates [3, 17]. Study data of intrapartum stillbirths rate highlight problems with accessibility and quality of the health care system in the field of antenatal and obstetric care. Improvement needs in Latvian healthcare system were documented also from international organizations (European Commission and the World Bank). For instance, there is a need to consider developing clinical guidelines and pathways based on clear criteria and standardized methods; improve quality of service provision, and coordination of services among healthcare providers and emerging legislation and regulatory frameworks [26].

Further work should be done to analyse and audit intrapartum death cases to identify areas of obstetric care for improvement. In 2017 improvements have been made in obstetric care, for instance, defined a procedure for the identification of high-risk patients and risk management; action plan and management of care in cases with common childbirth complications; obligatory maternal and perinatal audit in medical institutions etc.) [27].

The main strength of the study includes the fact that the data were population-based. This kind of epidemiological data is essential for health care planning and for determining temporal trends. The limitation is the lack of a comparison group of live births, which could be useful to determine other risk factors of LFD. Future research must focus on the causes of stillbirth. The results of this study may deserve attention for policy implementation regarding strategies to improve antenatal and obstetric labour and delivery care for women, in order to substantially reduce the number of stillbirths and strengthen perinatal audits at the national level.

## 5. Conclusions

The overall LFD rate showed a slight statistically significant reduction (p < 0.001) over the study periods (2001-2007 and 2008-2014). Intrapartum LFD slightly decreased (RR 0.6/1000 births). Substantial intrapartum stillbirths rates indicate problems with quality of intrapartum care and emergency obstetric care. Further research is needed to evaluate the strategies necessary to essentially reduce the number of stillbirths in the country and to provide detailed analysis of LFD causes. Improvement in female literacy, health education, identifying high-risk pregnancies, and periodic audits of all stillbirths can help in reduction of stillbirths.

## Figures and Tables

**Table 1:** Comparison of maternal and neonatal characteristics of LFD in 2001-2007 and 2008-2014.

**Characteristics**	**2001-2007**	**2008-2014 **	**p value**
	No (% (95CI))	No (% (95CI))	
Mean maternal age^1^	28 (23-33)	28 (24-33)	NS
Mean gestational week^1^	36 (32-39)	36 (32-39)	NS
Mean birth weight (g)^1^	2340 (1660-3046)	2405 (1550-3170)	NS
Antepartum	568 (77.2 (74.0-80.1))	503 (83.3 (80.2-86.1))	<0.001
Intrapartum	168 (22.8 (19.9-26.0))	101 (16.7 (13.9-20.0))	<0.001
*Maternal age groups* ^*2*^			
≤19 years	65 (8.8 (7.0-11.1))	38 (6.3 (4.6-8.4))	NS
20-34 years	521 (70.8 (67.6-74.2))	439 (72.7 (69.0-76.1))	NS
≥35 years	150 (20.4 (17.6-23.5))	127 (21.0 (17.9-24.4))	NS
*Gestational age, weeks* ^*2*^			
*28 – 31 weeks*	158 (21.5 (18.6-24.5))	130 (21.5 (18.4-25.0))	NS
*32 – 36 weeks*	247 (33.6 (30.2-37.0))	197 (32.6 (29.0-36.4))	NS
*≥37 weeks*	331 (45.0 (41.4-48.6))	277 (45.9 (42.0-50.1))	NS
*Birth weight groups, g* ^*2*^			
500-1499 g	140 (19.0 (16.3-22.0))	140 (23.2 (19.9-26.7))	NS
1500-2499 g	270 (36.7 (33.3-40.2))	173 (28.6 (25.1-32.4))	NS
≥2500 g	326 (44.3 (40.7-47.9))	291 (48.2 (44.2-52.2))	NS
Multiple births^2^	33 (4.5 (3.2-6.2))	40 (6.6 (4.8-8.8))	NS
Late first antenatal visit (after 12 GW)^2^	257 (34.9 (31.5-38.4))	132 (21.9 (18.7-25.3))	<0.001
Smoking during pregnancy^2^	162 (22.0 (19.1-25.1))	110 (18.2 (15.3-21.4))	NS
Foetal growth restriction^2^	24 (3.3 (2.1-4.7))	27 (4.5 (3.0-6.3))	NS
Pregnancy-induced hypertension^2^	39 (5.3 (3.8-7.1))	35 (5.8 (4.1-8.0))	NS
Preeclampsia^2^	46 (6.3 (4.7-8.2))	41 (6.8 (5.0-9.0))	NS
Placenta abruption^2^	56 (7.6 (5.9-9.7))	21 (3.5 (2.2-5.2))	<0.001

^1^Represents median (25th and 75th percentile) and Mann-Whitney U test is used.

^2^Represents n (% (95% CI)) and Chi-square test is used; NS: Not Significant.

**Table 2:** The birth and LFD statistics in 2001-2007 and 2008-2014.

	**2001-2007**	**2008-2014**	**Rate ratio (95**%** CI)**
Total birth number	147365	143466	
Number of live births	147573	144043	
Number of LFD	736	604	
LFD^*∗*^	5.0	4.2	0.8 (0.2-1.9)
LFD antepartum	3.8	3.5	0.9 (0.9-1.2)
LFD intrapartum	1.1	0.7	0.6 (0.3-1.0)
LFD by multiple births	10.2	11.3	1.1 (0.6-1.9)
LFD by maternal age:			
≤19 years	4.7	4.5	1.0 (0.3 - 2.4)
20-34 years	4.4	3.9	0.9 (0.2 - 2.0)
≥35 years	9.2	5.8	0.6 (0.2 - 1.2)

^*∗*^LFD (late foetal death) rates were calculated per 1000 total births (live and still).

## Data Availability

The data used to support the findings of this study were provided by The Centre for Disease Prevention and Control (CDPC) of Latvia under license and so cannot be made freely available. Access to these data will be considered by the author upon request with permission.

## Additional Points

This work was supported by National Research Programme Biomedicine for Public Health (BIOMEDICINE). Research on acute and chronic diseases in a wide age range of children helps to develop diagnostic and therapeutic algorithms to reduce mortality, prolong survival, and improve quality of life.
